# Two‐step changes in paced QRS morphology suggest capturing at different levels of posterior fascicle fibers during left bundle branch area pacing: A case report

**DOI:** 10.1002/joa3.12882

**Published:** 2023-06-12

**Authors:** Takashi Okajima, Shinji Ishikawa, Yusuke Uemura, Kenji Takemoto, Masato Watarai

**Affiliations:** ^1^ Department of Cardiology Anjo Kosei Hospital Anjo Japan

**Keywords:** conduction system pacing, left bundle branch area pacing, left bundle posterior fascicle, Purkinje network, QRS morphology

## Abstract

Two‐step changes in paced QRS morphology during the left bundle branch area pacing threshold test. It suggests that capturing occurs at multiple sites of the left bundle branch‐Purkinje system.

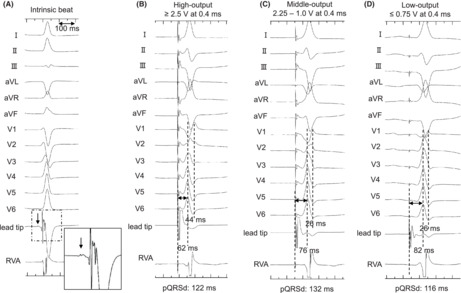

Left bundle branch (LBB) area pacing (LBBAP) is widely performed as conduction system pacing because it has a lower threshold and is technically easier than His bundle pacing. Although LBBAP can capture the LBB–Purkinje conduction system and create a narrow‐paced QRS duration (pQRSd), several different capture types and paced QRS morphologies are observed. Recently, left bundle fascicular (LBF) capture has been reported to be the predominant type of LBBAP in clinical practice.^1^ However, LBB–Purkinje potentials are not often identified, and the mechanism of creating a paced QRS morphology based on different conduction system capturing has not been fully understood.

A 69‐year‐old woman presented with bradycardia and dyspnea on exertion. An electrocardiogram showed a complete or two‐to‐one atrioventricular block. Her heart contractions were normal without any features of cardiomyopathy. After informed consent was obtained, we planned to implant an LBBAP.

First, we performed His bundle potential (HBP) mapping using the C315 His sheath and select secure™ 3830 lead (Medtronic, Inc.) as an angiographic marker. Subsequently, the 3830 lead was used as the LBBAP lead and screwed on the ventricular septum with a well‐fixed sheath contact approximately 2.4 cm apical from the HBP‐recorded site. We performed deep screwing with terminal monitoring of pQRSd and stimulus‐to‐left ventricular activation time (Stim‐LVAT) in lead V6. The pQRSd and Stim‐LVAT were gradually shortened. Finally, the pQRSd and Stim‐LVAT at the deep site of the septum were 122 and 62 ms, respectively. We assumed that the lead reached the LBB area (Figure 1). An isolated small potential that was 18 ms earlier than the QRS onset and a spiky potential, which was observed before local ventricular excitation, were identified at the site (Figure 2A).

**FIGURE 1:**
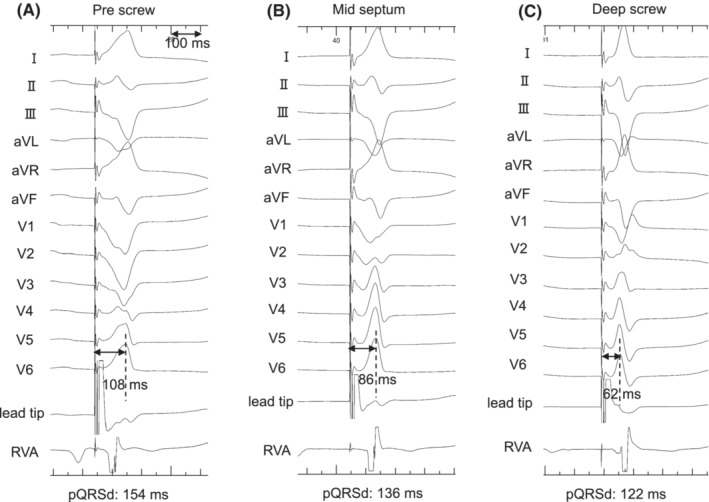
The changes of paced electrocardiograms during deep screwing of LBBAP lead. Twelve lead electrocardiogram and intracardiac electrocardiogram of LBBAP lead tip unipolar electrode and the bipolar electrode of 5076 lead used for ventricular backup pacing. (A) Pacing at the right ventricle septum pre‐screwing showed that the pQRSd was 154 ms and the Stim‐LVAT was 108 ms with W‐type morphology of V1 lead. (B) After the lead advanced to the mid‐septum, pacing showed that the pQRSd was 130 ms and the Stim‐LVAT was 86 ms. (C) After additional lead screwing, the pacing showed that the pQRSd was 122 ms and the Stim‐LVAT was 62 ms with QR morphology of V1 lead. It was indicated that the LBBAP lead tip was reached in the LBBA. LBBA, left bundle branch area; LBBAP, left bundle branch area pacing; pQRSd, paced QRS duration; RVA, right ventricular apex; Stim‐LVAT, stimulus‐to‐left ventricular activation time.

**FIGURE 2:**
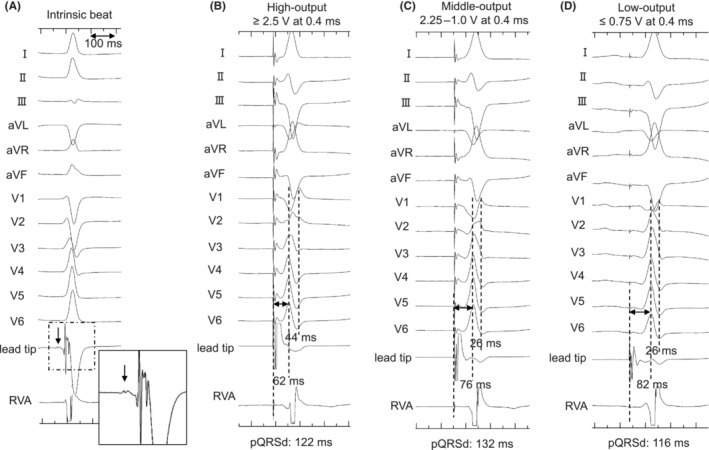
Paced QRS morphology changes during threshold test from the LBBAP lead unipolar pacing. Twelve lead electrocardiogram and intracardiac electrocardiogram of LBBAP lead tip unipolar electrode and the bipolar electrode of 5076 lead used for ventricular backup pacing. (A) During the intrinsic beat of two to one atrioventricular block, the isolated small potential was observed 18 ms early from QRS onset, and the spiky potential was observed before local ventricular excitation. (B) With high‐output pacing of ≥2.5 V at 0.4 ms, the Stim‐LVAT was 62 ms, the V6‐V1 interpeak interval was 44 ms, and the V1 morphology was QR pattern. (B) With middle‐output pacing of 2.5–1.0 V at 0.4 ms, the Stim‐LVAT was 76 ms, the V6‐V1 interpeak interval was 26 ms, and the V1 morphology was Qr pattern. (C) With low‐output pacing of ≤0.75 V, a 32‐ms isoelectric interval was observed after the pacing spike artifact, the Stim‐LVAT was 82 ms, the V6‐V1 interpeak interval was 26 ms, the V1 morphology was qr pattern, and the time from stimulus to QRS end was prolonged as 148 ms. LBBAP, left bundle branch area pacing; pQRSd, paced QRS duration; RVA, right ventricular apex; Stim‐LVAT, stimulus‐to‐left ventricular activation time.

Two‐step abrupt QRS morphological changes were observed during the threshold test using unipolar pacing from the lead tip electrode (Figure 2B–D). The first step change was observed from the high‐output pacing of ≥2.5 V at 0.4 ms to the middle‐output pacing of 2.5–1.0 V. Specifically, Stim‐LVAT was prolonged from 62 to 76 ms, V6‐V1 interpeak interval^2^ was shortened from 44 to 26 ms. The second QRS morphology change was observed from the middle to low‐output pacing of ≤0.75 V. A 32‐ms isoelectric interval appeared after the pacing spike artifact, and the V1 lead morphology was changed from a Qr pattern to a qr pattern. Moreover, the time from stimulus‐to‐QRS end was prolonged from 132 to 148 ms. The QRS axis showed the left‐axis deviation for all outputs, although there were slight differences. We believe that the pQRSd and Stim‐LVATs were sufficiently short during all outputs, and the operation was completed. Echocardiography was used to confirm the LBBAP lead position near the left ventricular septum endocardium (Figure 4).

Two‐step QRS morphology changes based on pacing output can be observed during the threshold test of LBBAP. A possible mechanism of this unusual observation is that unipolar pacing from the LBBAP lead tip was captured at multiple sites of the LBB–Purkinje system and/or left ventricular septal (LVS) myocardium. A previous report showed that the paced QRS axis changes depended on the pacing output.^3^ In the present case, the paced QRS axis retained left‐axis deviation in all outputs. Furthermore, the QRS morphology changed abruptly, the Stim‐LVAT was prolonged by 14 ms, and the V6‐V1 interpeak interval was shortened by 18 ms from the high to the middle output. These findings indicate that non‐selective left bundle posterior fascicle (LBPF) pacing was created by the high output.^2^, ^4^ From the middle to the low output, a secondary abrupt change was observed with a 32‐ms isoelectric interval and prolonged interval of stimulus‐to‐QRS end. This indicated that there was a loss of capture at the LVS, and that the distal fiber of the LBB–Purkinje system was selectively captured by the low‐output pacing (Figure 3). The prolonged time from stimulus to the whole ventricular excitation might be affected by the retrograde conduction time from the captured distal fiber of LBB–Purkinje to LBPF. Moreover, the small potential in the intrinsic beat was suspected to be the LBPF potential, and the spiky potential was suspected to be the distal LBB–Purkinje potential. Captured LBF could be categorized based on the paced QRS axis when each fascicle was selectively captured.^1^ Further detailed observation in this case revealed slightly stronger positive components in the lead II and aVF at the middle‐output pacing rather than the high‐output pacing, compared to the low‐output pacing. We supposed that selective LBF captures were not obtained under the high and middle‐output pacing. Therefore, the small positive components in the inferior lead might be created by the directly captured LVS excitations. In the present study, however, we could not identify retrograde HBP or LBBP during the pacing test, which is a limitation of this hypothesis.

**FIGURE 3:**
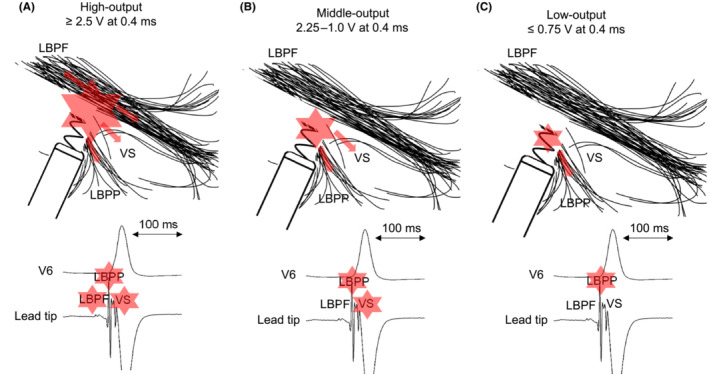
Schemas showing that the captured area depends on the outputs. The intrinsic beats' potentials suspected to be captured depend on the output as shown below. Red stars represented the captured areas and potentials. (A) The high‐output pacing captured LBPF, VS, and LBPP. (B) The middle‐output pacing captured VS and LBPP. (C) The low‐output pacing captured LBPP. LBPF, left bundle posterior fascicle; LBPP, left bundle posterior Purkinje; VS, ventricular septum.

**FIGURE 4:**
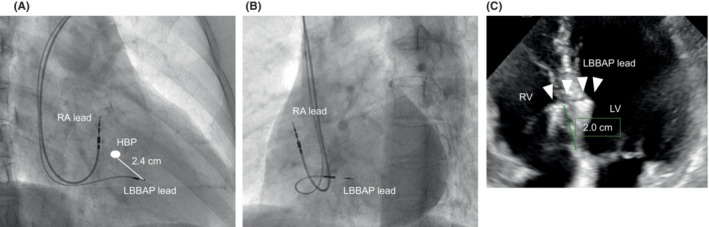
Final fluoroscopic images showing the lead location at (A) an RAO of 30 degrees and (B) an LAO of 50 degrees. (C) Echocardiogram showing the location of the LBBAP lead in the ventricular septum. The LBBAP lead was located approximately 2.0 cm apical from the membranous septum, and the lead tip was near the left ventricular septum endocardium. HBP, His bundle potential; LAO, left anterior oblique; LBBAP, left bundle branch area pacing; LV, left ventricle; RA, right atrium; RAO, right anterior oblique; RV, right ventricle.

In clinical practice, some cases in which the LBBAP is considered successful could have distal LBB–Purkinje system pacing. A previous study showed that the success rate of strict LBBP was approximately 30% for LBBAP.^2^ The LBB–Purkinje system, especially the posterior fascicular area, has been reported to have individual differences and complex networks in the distal site that spread as a broad sheet‐like structure.^5^ The proximal and distal sites of the conduction system may be anatomically close. Therefore, even if the LBBAP lead is placed in anatomically similar sites, the capture level of the LBB–Purkinje system may change on a case‐by‐case basis. It is unclear whether it is better to capture a more central site or whether any conduction system would be equally effective in long‐term outcomes, especially in heart failure cases or in cases where cardiac resynchronization therapy efficacy is expected.

In conclusion, this is the first report of two‐step paced QRS morphology changes during the LBBAP threshold test, which suggests that capturing occurs at multiple sites of the LBB–Purkinje system. This may be due to the complex networks of LBPF regions. Some cases of LBBAP might include capturing a distal site of the LBB–Purkinje system.

## CONFLICT OF INTEREST STATEMENT

The authors have nothing to disclose.

## ETHICS APPROVAL STATEMENT

Approval was obtained from the local ethics committee.

## INFORMED CONSENT

The authors obtained consent from the patients.

## REGISTRY AND THE REGISTRATION NO.

N/A.

## ANIMAL STUDIES

N/A.

## Data Availability

Available upon reasonable request.

## References

[1] Jastrzębski M , Kiełbasa G , Cano O , Curila K , Heckman L , De Pooter J , et al. Left bundle branch area pacing outcomes: the multicentre European MELOS study. Eur Heart J. 2022;43(40):4161–73. 10.1093/eurheartj/ehac445 35979843 PMC9584750

[2] Jastrzębski M , Burri H , Kiełbasa G , Curila K , Moskal P , Bednarek A , et al. The V6‐V1 interpeak interval: a novel criterion for the diagnosis of left bundle branch capture. Europace. 2022;24(1):40–7. 10.1093/europace/euab164 34255038 PMC8742628

[3] Iida Y , Makishima N . Uncommon output‐dependent paced QRS morphology transition during left bundle branch pacing. Pacing Clin Electrophysiol. 2022;45(10):1229–32. 10.1111/pace.14516 35598105

[4] Wu S , Chen X , Wang S , Xu L , Xiao F , Huang Z , et al. Evaluation of the criteria to distinguish left bundle branch pacing from left ventricular septal pacing. JACC Clin Electrophysiol. 2021;7(9):1166–77. 10.1016/j.jacep.2021.02.018 33933414

[5] Syed FF , Hai JJ , Lachman N , DeSimone CV , Asirvatham SJ . The infrahisian conduction system and endocavitary cardiac structures: relevance for the invasive electrophysiologist. J Interv Card Electrophysiol. 2014;39(1):45–56. 10.1007/s10840-013-9858-7 24322419 PMC4456398

